# Microarray analysis of lung long non-coding RNAs in cigarette smoke-exposed mouse model

**DOI:** 10.18632/oncotarget.23362

**Published:** 2017-12-18

**Authors:** Hao Wang, Lei Chen, Diandian Li, Ni Zeng, Yanqiu Wu, Tao Wang, Yongchun Shen, Dan Xu, Fuqiang Wen

**Affiliations:** ^1^ Department of Respiratory and Critical Care Medicine, West China Hospital of Sichuan University, and Division of Pulmonary Diseases, State Key Laboratory of Biotherapy of China, Chengdu 610041, China

**Keywords:** long non-coding RNAs, cigarette smoke, airway inflammation, mice, microarray analysis

## Abstract

Several studies have demonstrated the function of long nonâ€‘coding RNAs (lncRNAs) in various biological processes, yet their role underlying the susceptibility to cigarette smoke (CS)-induced airway inflammation remains limited. In the present study, we aimed to profile the expression of lncRNAs and mRNAs in CS-exposed mice. C57BL/6 mice were assigned into a single cigarette-smoking machine with or without CS exposure for 4 weeks, followed by lung tissue harvest and RNA isolation. Microarray analysis identified 108 lncRNAs and 119 mRNAs with differential expression levels in CS-exposed mouse lung tissue compared with those in control mice. The expression patterns of several lncRNAs were further confirmed by qRT-PCR. GO and pathway analyses showed that the altered mRNAs were mainly related to the processes of immune response, defense response and cell chemotaxis, cytokine-cytokine receptor interaction and chemokine signaling pathway. Moreover, a single lncRNA may co-expressed with several mRNAs, and so was the mRNA. Our findings uncovered the expression profile of lncRNAs and mRNAs in the lungs of CS-exposed mice, which may offer new insights into pathogenesis of CS-associated airway inflammatory disorders.

## INTRODUCTION

Tobacco use, primarily associated with cigarette smoking, is a worldwide risk factor of public health. Cigarette smoke (CS) contains over 7000 toxicants, most of which are etiological factors in the development of inflammatory pulmonary diseases. Chronic CS exposure causes damage to lung resident cells such as airway epitheliums, leading to the release of pro-inflammatory cytokines and the recruit of neutrophils, contributing to airway remodeling and subsequent airflow limitation which has been identified as the prominent feature of chronic obstructive pulmonary disease (COPD) or other CS-related airway inflammatory disorders [1, 2]. However, previous studies found that neutrophilic airway inflammation already occurred upon short-term CS exposure in susceptible mice and humans [3, 4], indicating that genetic or epigenetic factors may play a role in the susceptibility to CS-induced airway inflammation.

Long non-coding RNAs (lncRNAs), as a class of non-coding RNAs (ncRNAs), is generally defined as transcripts of greater than 200 nucleotides. As previously considered unfunctional, lncRNAs are now believed to be involved in various biologic processes, including inflammation, oxidative stress, cell growth and apoptosis at both transcriptional and posttranscriptional levels [5–7]. Several studies demonstrated the function of lncRNAs in the development of pulmonary disorders related to CS. In human bronchial epithelial cells (HBEs), the lncRNA, Hox transcript antisense intergenic RNA (HOTAIR), was found to be correlated with cigarette smoke extract (CSE)-induced changes in cell cycle, while the knockdown of cancer-associated lncRNA-1 (SCAL1) in HBEs showed a significant potentiation of the cytotoxicity induced by CSE [8]. Besides, hundreds of differentially expressed lncRNAs were found in the lung tissues of healthy smokers compared with those in non-smokers [9], indicating that CS exposure may regulate the expression of lncRNAs, leading to the development of CS-induced lung disorders. However, genetics regarding to lncRNAs underlying the susceptibility to CS-induced airway inflammation remains limited.

Our previous research, which described a CS-exposed mouse model system, found that four-week CS-exposed mice could recapitulate the morphological and functional changes of human chronic airway inflammation, including increased levels of pro-inflammatory cytokines and inflammatory cell counts in bronchoalveolar lavage fluid (BALF), thickening of the airway epithelium, and peribronchial inflammatory cell infiltration [10]. Based on this model, in the present study, we aimed to profile the expressions of lncRNAs and mRNAs in the lung tissues of CS-exposed mice by microarray analysis.

## RESULTS

### LncRNA and mRNA expression profile

**Figure 1:**
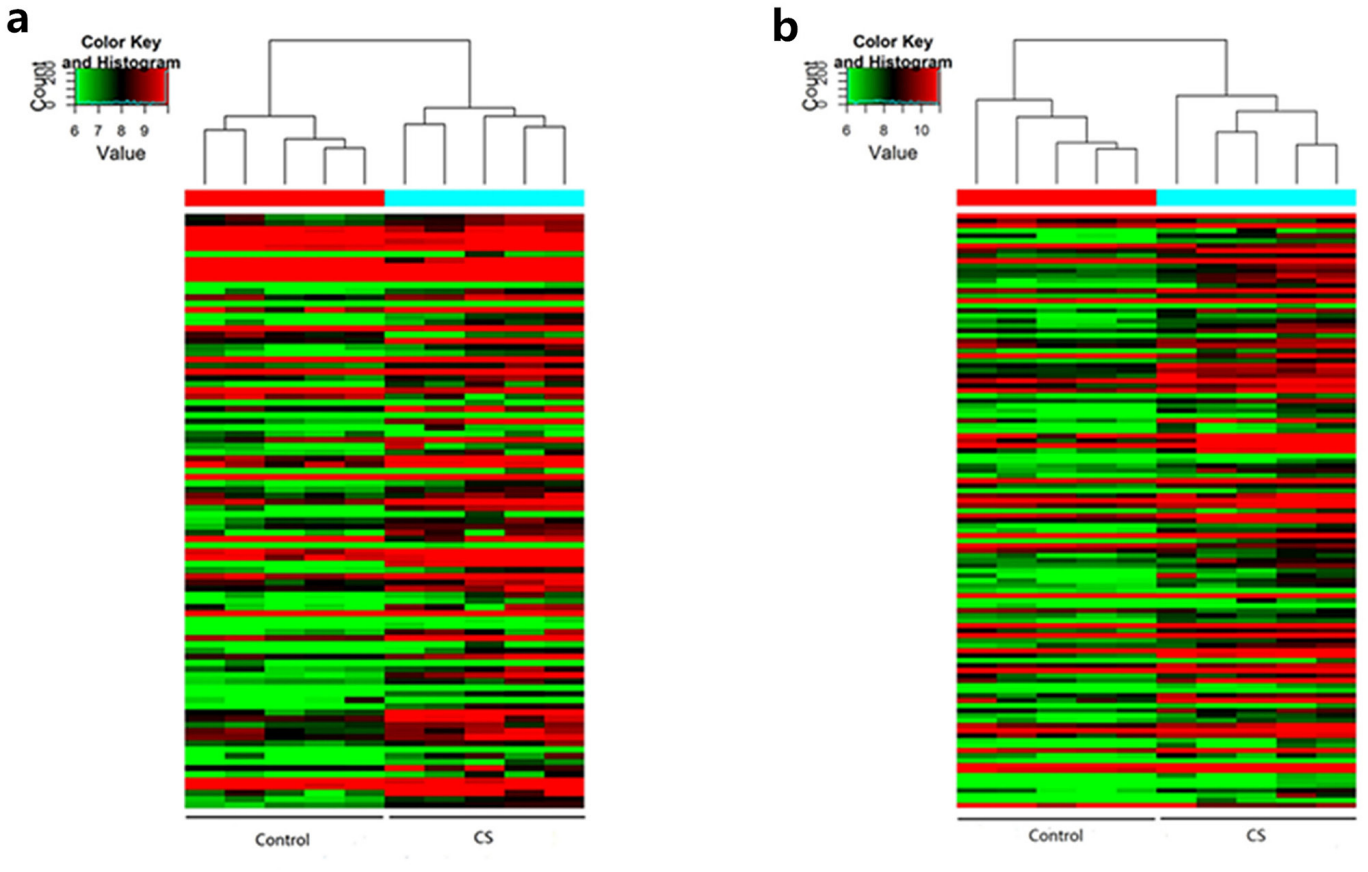
Heat maps showing the distinct lncRNA **(a)** and mRNA **(b)** expression profiles between CS-exposed mice and control mice. Notes: Hierarchical clustering of significantly (P < 0.05, >2-fold change) regulated lncRNAs (a) and mRNAs (b) are shown as heat maps. Expression values are represented with different colors ranged from green to red, indicating low relative expression to high relative expression, respectively. (n=5 for control group and CS-exposed group, respectively). CS: cigarette smoke-exposed group.

As shown in Figure 1, 108 lncRNAs (Figure 1a) and 119 mRNAs (Figure 1b) were detected with significantly differential expression levels in CS-exposed mouse lung tissue when comparing with controls (fold-change >2.0; *P* < 0.05). Among these lncRNAs, AK020757 (fold-change: 39.25, *P* = 8.97E-07) and AK085915 (fold-change: 3.99, *P* = 0.0032) were the most up- and down-regulated lncRNAs in CS-exposed mice, respectively. While for mRNAs, Gm12429 (fold change: 18.26, *P* = 1.59E-08) and apelin receptor (Aplnr) (fold change: 2.97, *P* = 0.0023) were the most up- and down-expressed, respectively. The top 20 differentially expressed lncRNAs and mRNAs identified by microarray analysis are listed in Tables 1 and 2, respectively.

**Table 1:** The detailed information of the top 10 up-regulated and top 10 down-regulated lncRNAs

lncRNA name	Regulation direction	Normalized intensity		Fold change	P-value
		CON group	CS group		
AK020757	up	4.93±0.71	10.23±0.52	39.25	8.97E-07
AK047207	up	7.49±0.61	12.51±0.34	32.43	2.21E-07
uc029vco.1	up	3.63±1.00	7.86±0.66	18.72	4.87E-05
uc007pgi.1	up	10.57±0.98	14.63±1.40	16.68	0.0007
uc029slj.1	up	3.63±0.69	7.29±0.54	12.60	1.47E-05
AK076311	up	6.25±0.08	9.30±0.59	8.32	3.19E-06
ENSMUST00000107991	up	6.12±0.83	9.13±0.44	8.04	9.70E-05
uc029usn.1	up	9.00±0.49	12.00±0.48	8.01	5.04E-06
ENSMUST00000178906	up	9.41±0.46	12.38±0.48	7.85	8.27E-06
uc007coi.2	up	3.49±0.50	6.17±0.95	6.45	0.0005
AK085915	down	8.39±0.49	6.39±0.96	3.99	0.0032
TCONS_00004206	down	12.29±0.35	10.48±0.95	3.50	0.0041
ENSMUST00000144634	down	9.27±0.35	7.58±1.10	3.23	0.0113
ENSMUST00000181247	down	11.69±0.53	10.26±0.53	2.69	0.0027
ENSMUST00000145428	down	5.77±0.37	4.45±0.93	2.49	0.0187
ENSMUST00000155715	down	10.34±0.28	9.04±0.84	2.47	0.0111
NR_045048	down	12.22±0.21	10.98±0.46	2.35	0.0006
ENSMUST00000151765	down	6.48±0.99	5.28±0.53	2.29	0.0449
TCONS_00015067	down	6.15±0.52	4.98±0.51	2.25	0.0071
NR_037693	down	6.95±0.48	5.78±1.01	2.25	0.0472

The name, regulation direction, normalized intensity, fold change and *p*-value of the top 10 up-regulated and top 10 down-regulated lncRNAs between cigarette smoke-exposed mice and controls, for the normalized intensity, data were presented with mean± standard deviation. CON: control; CS: cigarette smoke.

**Table 2:** The detailed information of the top 10 up-regulated and top 10 down-regulated mRNAs

Gene symbol	Description	Regulation direction	Normalized intensity		Fold change	P-value
			CON	CS		
Gm12429	Predicted gene 12429	up	3.01±0.17	7.20±0.38	18.26	1.59E-08
Igj	Immunoglobulin joining chain	up	7.84±0.94	11.53±1.09	12.90	0.0004
Camp	Cathelicidin antimicrobial peptide	up	11.22±1.65	14.86±0.89	12.47	0.0024
1700112E06Rik	RIKEN cDNA 1700112E06 gene	up	7.82±0.54	11.12±0.50	9.83	8.03E-06
Cxcl9	Chemokine (C-X-C motif) ligand 9	up	4.15±0.83	7.35±1.55	9.23	0.0036
Fcnb	Ficolin B	up	4.27±1.60	7.41±0.92	8.81	0.0052
S100a9	S100 calcium binding protein A9	up	7.46±0.74	10.42±1.03	7.78	0.0008
Cd177	CD177 antigen	up	7.72±1.19	10.66±0.35	7.68	0.0007
Gm5416	Predicted gene 5416	up	4.90±0.85	7.68±0.83	6.88	0.0008
4933402N22Rik	RIKEN cDNA 4933402N22 gene	up	6.64±0.48	9.25±0.97	6.09	0.0007
Aplnr	Apelin receptor	down	9.44±0.27	7.87±0.75	2.97	0.0023
Vmn2r122	Vomeronasal 2, receptor, 122	down	6.11±0.35	4.63±0.56	2.79	0.0010
Fabp1	Fatty acid binding protein 1, liver	down	13.13±0.29	11.69±0.46	2.71	0.0004
Sprr1a	Small proline-rich protein 1A	down	11.25±0.67	9.88±0.67	2.58	0.0122
Heph	Hephaestin	down	5.45±0.74	4.11±1.04	2.54	0.0468
Slurp1	Secreted Ly6/Plaur domain containing 1	down	11.25±0.54	9.96±0.69	2.44	0.0112
Fbn2	Fibrillin 2	down	9.20±0.49	7.99±0.50	2.32	0.0047
Tet1	Tet methylcytosine dioxygenase 1	down	5.49±0.52	4.29±0.83	2.31	0.0251
Tet1	Tet methylcytosine dioxygenase 1	down	10.94±0.34	9.78±0.61	2.23	0.0059
Egfbp2	Epidermal growth factor binding protein type B	down	10.69±0.38	9.57±0.40	2.16	0.0020

The Gene symbol, description, regulation direction, normalized intensity, fold change and *p*-value of the top 10 up-regulated and top 10 down-regulated mRNAs between cigarette smoke-exposed mice and controls, for the normalized intensity, data were presented with mean± standard deviation. CON: control; CS: cigarette smoke.

### qRT-PCR validation

**Figure 2:**
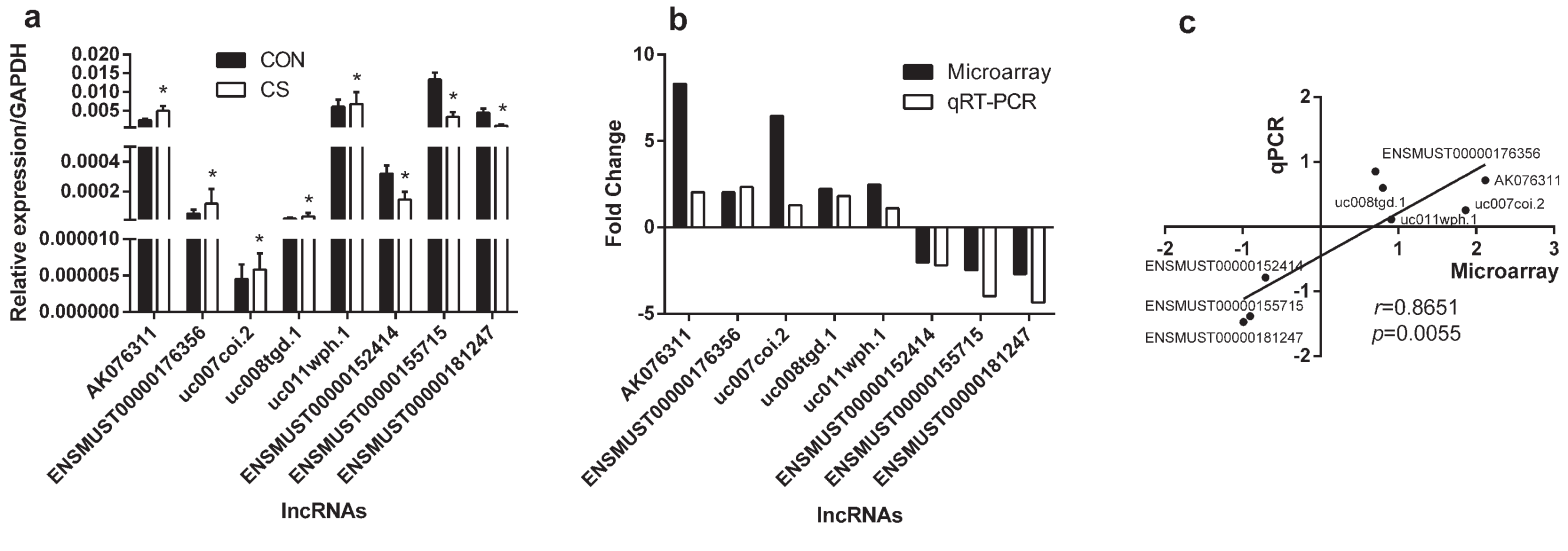
Comparison between microarray data and qRT-PCR results qRT-PCR was performed to test the differentially expressed lncRNAs between controls and CS-exposed mice **(a)**, the fold change of each lnRNA between CS-exposed mice and controls was tested with microarray and qRT-PCR respectively **(b)**, and the correlation between microarray and qRT-PCR was performed with natural logarithms of these different fold changes **(c)**. ^*^: *p*<0.05, *r*: standard correlation coefficient. (n=5 for control group and CS-exposed group, respectively). CON: control group; CS: cigarette smoke-exposed group.

To validate the reliability of the microarray results and determine the role of lncRNAs in CS-exposed mice, 8 differentially expressed lncRNAs were randomly selected and analyzed by quantification real-time PCR (qRT-PCR). As shown in Figure 2a, five of these lncRNAs were up-expressed in CS-exposed mice when comparing with those in control mice, and three were down-regulated. Besides, the fold-changes of these up-regulated and down-regulated lncRNAs were calculated with microarray or qRT-PCR (Figure 2b), and the correlation analysis showed that the results of microarray were highly positively correlated with qRT-PCR (Figure 2c), suggesting that our microarray data was reliable.

### GO and KEGG pathway analyses

**Figure 3:**
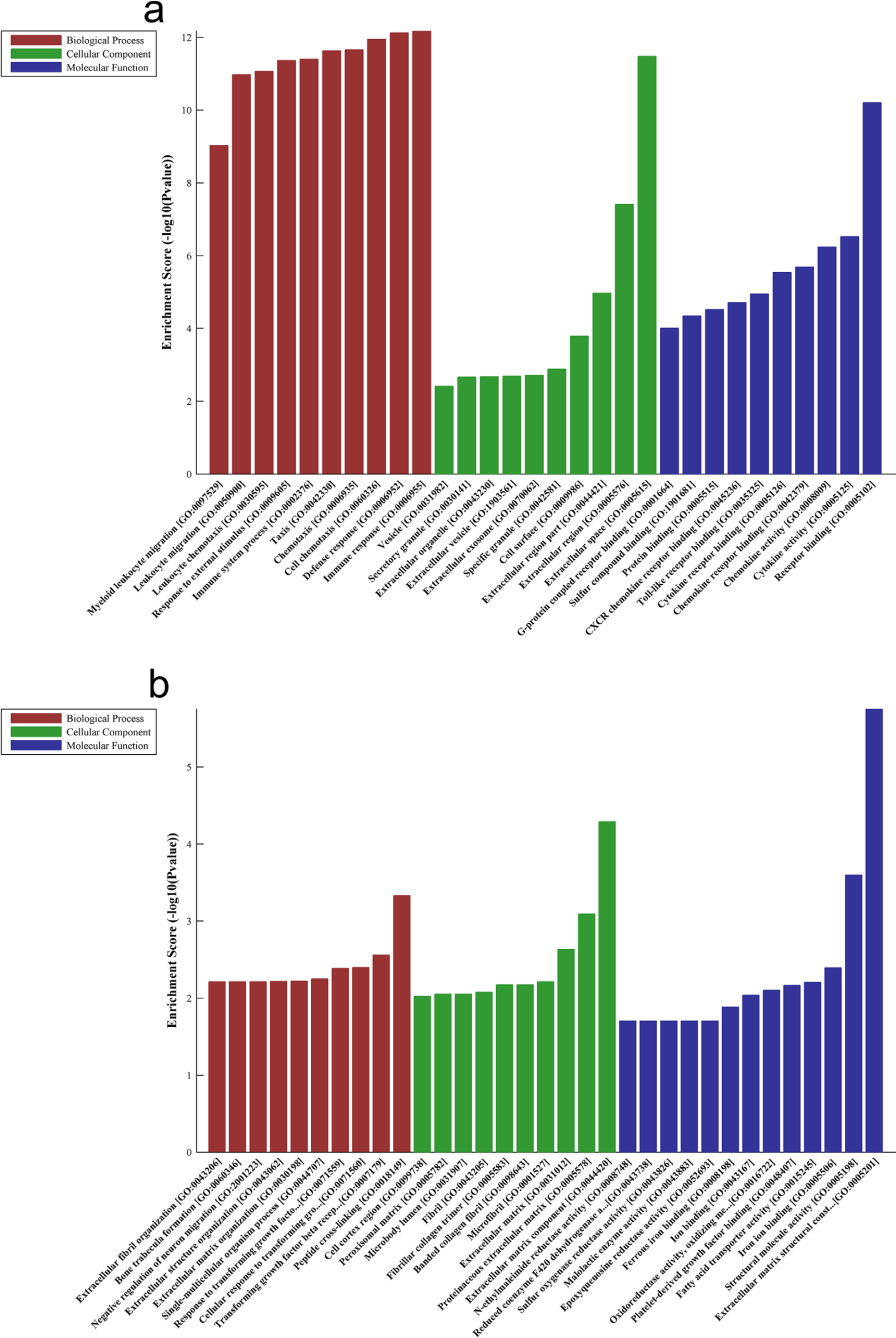
Biological functions of up-expressed and down-expressed mRNAs **Notes:** The most significantly up-regulated mRNAs **(a)** and down-regulated mRNAs **(b)** which were involved in biological process, cellular component and molecular function were achieved with GO analysis.

The Gene Ontology (GO) results showed that the most significant enriched biological processes of up-regulated genes were immune response, defense response and cell chemotaxis (Figure 3a), the most significant enriched cellular components of up-regulated genes were extracellular space, extracellular region and extracellular region part (Figure 3a), and the most significant enriched molecular function of up-regulated genes were receptor binding, cytokine activity and chemokine activity (Figure 3a). On the other hand, the most significant enriched biological processes of down-regulated genes were peptide cross-linking, transforming growth factor beta receptor signaling pathway and cellular response to transforming growth factor beta stimulus (Figure 3b), the most significant enriched cellular components of down-regulated genes were extracellular matrix component, proteinaceous extracellular matrix and extracellular matrix (Figure 3b), and the most enriched GOs targeted by down-regulated transcripts were extracellular matrix structural constituent, structural molecule activity and iron ion binding (Figure 3b).

**Figure 4:**
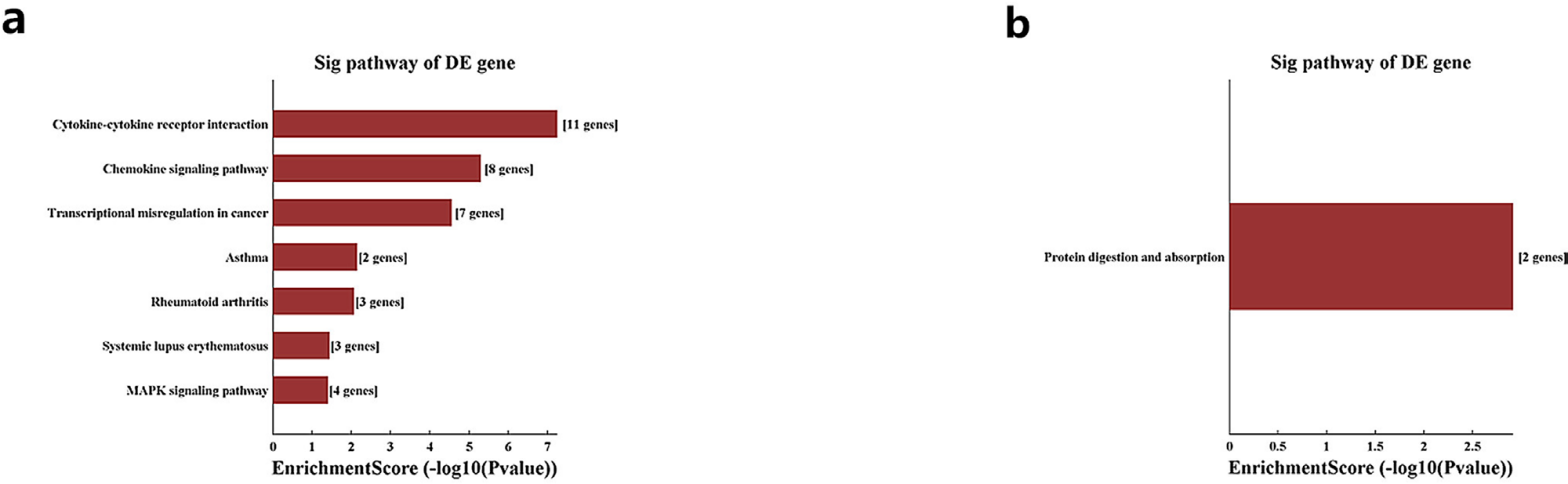
Pathway analysis for up-regulated and down-regulated mRNAs **Notes:** The most significant pathways which were related to the up-regulated genes **(a)** and down-regulated genes **(b)** were achieved with pathway analysis.

Pathway analysis demonstrated that the up-regulated genes are mainly associated with cytokine-cytokine receptor interaction and chemokine signaling pathway, while down-regulated transcripts in CS-treated lung tissues are involve in protein digestion and absorption (Figure 4).

### LncRNA-mRNA co-expression network

**Figure 5:**
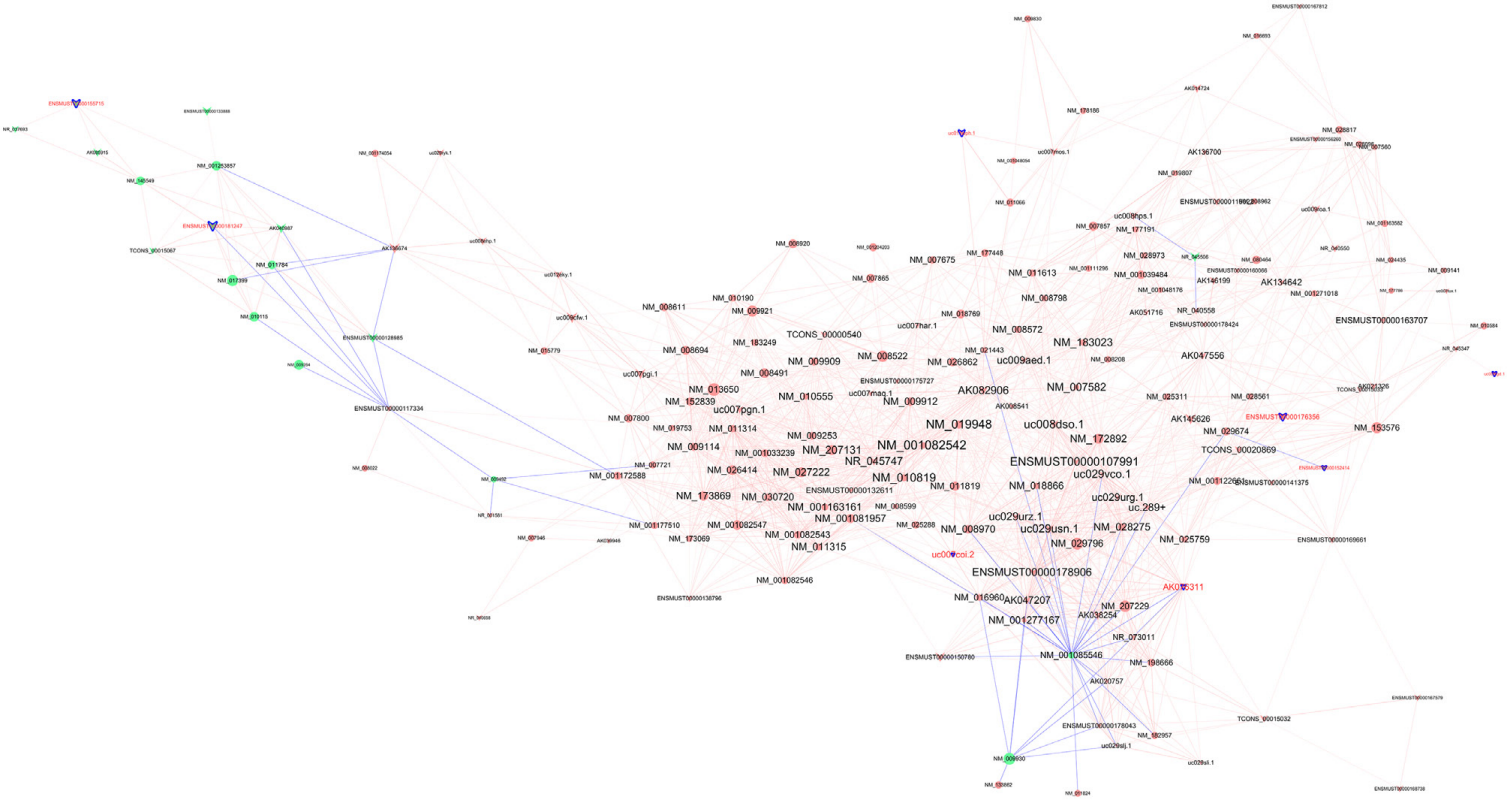
Co-expression network of the differentially expressed lncRNAs and mRNAs **Notes:** Round nodes represent protein-coding genes and arrow nodes represent lncRNAs. Red nodes represent up-regulated genes or lncRNAs, green nodes represent down-regulated genes or lncRNAs, and blue nodes represent those 8 tested lncRNAs. A red line represents a positive correlation, and a blue line represents negative correlation. The node size represents the connectivity, briefly, larger node means that more genes or lncRNAs are co-expressed with this gene or lncRNA.

An lncRNA-mRNA co-expression network was constructed, all the differentially expressed lncRNAs which were related to mRNAs with a pearson’s correlation coefficients (PCC) of no less than 0.9 were shown in the same figure, and the validated 8 lncRNAs were marked as blue (Figure 5). Most lncRNAs including the 8 ones were co-expressed with multiple mRNAs and lncRNAs, indicating that multiple trans-regulative mechanisms were present.

## DISCUSSION

Recently, growing investigations on transcriptome sequencing have evealed thousands of differentially expressed lncRNAs in various diseases, which may play roles in the development and progression of diseases [11–13]. However, studies focusing on the lncRNAs related to CS-induced airway inflammation is limited. In this study, we investigated the lncRNA expression profiles in the lung tissue of 5 CS-exposed mice and 5 control mice to uncover the potential roles of lncRNAs in the pathogenesis of CS-induced pulmonary inflammation. As a result, 108 lncRNAs and 119 mRNAs were identified as differentially expressed via microarrays. Most of these lncRNAs have not been functionally characterized. The microarray results of AK076311, ENSMUST00000152414, ENSMUST00000155715, ENSMUST00000176356, ENSMUST00000181247, uc007coi.2, uc008tgd.1 and uc011wph.1 were confirmed by qRT-PCR. The data from qRT-PCR matched well with those from microarrays.

Previous reports have shown that lncRNAs participated in a wide variety of pathological processes at different levels, including regulation of gene transcription, chromatin remodeling and epigenetic regulation. Therefore, GO and pathway analyses were conducted to help better predicting on the potential function of the differentially expressed lncRNAs and co-expressed genes. The results showed that differentially expressed genes are mainly involved in immune response, defense response and cell chemotaxis. These genes were further identified to be mainly related to cytokine-cytokine receptor interaction and chemokine signaling pathways. At the early stages of COPD, airway inflammation is characterized by extensive activation of the innate immune system [14]. During this period, damage associated molecular patterns (DAMPs) induced by CS activate pattern recognition receptors (PRRs), leading to activation of inflammatory pathways and the release of inflammatory cytokines [15, 16]. These cytokines further induce the accumulation of innate immune cells to the damage site, along with the increasing production of chemokines [17]. Our results strongly supported the previous findings, implying that innate immune defense mechanism participates in the CS-induced airway inflammation and may offer clues for early intervention of COPD.

Moreover, from the coding-non-coding gene co-expression network (CNC network), we found that many lncRNAs were significantly correlated with the expression of multiple protein-coding genes. Notably, both AK076311 and uc007coi.2 were negatively associated with chemokine receptor 10 (CCR10), while ENSMUST00000181247 positively co-expressed with CD177. The involvement of CCR10 and its ligand CCL28 has been implicated in various inflammatory lung diseases [18]. CCL28 mediates *in vitro* T and B cell migration through CCR10 [19, 20]. In addition, it has been shown that CD177 levels were increased in the airways of Cynomolgus monkeys after ozone challenge, indicating the potential relevance with COPD pathophysiology [21, 22]. These findings are consistent with the GO and pathway analyses indicating the important role of innate immune response in CS-induced inflammation. Although according to current evidence, many lncRNAs may function locally to activate or repress the expression of their neighboring or overlapping genes [23], it is still worthy to perform further study to reveal the underlying mechanisms of these lncRNAs.

In conclusion, the present study profiled the expression of lncRNAs and mRNAs by microarray in the lung tissue of CS-exposed mice. Hundreds of lncRNAs and mRNAs were observed differentially expressed after CS exposure. GO and pathway analyses were made to speculate the potential functions of differentially expression genes. Further studies are required to clarify the molecular mechanism and biological function of lncRNAs to determine whether they can serve as novel targets in CS-associated airway inflammation.

## MATERIALS AND METHODS

### Animal experiments

Animals were handled-according to the ARRIVE guidelines developed by the National Center for the Replacement, Refinement, and Reduction of Animals in Research (NC3Rs), the study protocol was reviewed and approved by the animal ethics committee of West China Hospital, Sichuan University.

Specific pathogen-free male C57BL/6 mice (7-9 weeks, 22-24g) were purchased from Dashuo Biological Technology Co, Ltd (Chengdu, China), and were randomly divided into two groups (n =5 per group): control group (C) and CS-exposed group (CS).

Marlboro cigarette was employed for CS exposure experiment (Marlboro, Philip Morris USA Inc.; 0.8 mg nicotine and 10 mg tar per cigarette), the toxicity of which is similar to the 1R4F reference cigarette from University of Kentucky [24]. Mice in CS group were exposed to CS for 2 hours twice daily, 6 days per week for 4 weeks according to the protocol described previously [25]. Briefly, mice were assigned into a ventilated plastic chamber connected to a smoke generator (CH Technologies, Westwood, NJ, USA) and filled with fixed concentration of smoke (200 mg total particulate matter (TPM)/m^3^) by pumping mainstream cigarette smoke from burning cigarettes at a constant rate (each cigarette took 4 min to burn out) while using another pump to deliver fresh air from outside simultaneously at a fixed rate. The total rate of airflow passing through the box was constant at 1.22 L/min. At the same time, mice in control group were exposed to room air following the same schedules. When CS exposure finished, all the mice were sacrificed by over-dose of intraperitoneal phenobarbital (Sigma-Aldrich, St Louis, MO, USA) followed by lung tissue collection.

### RNA isolation

The total RNA from lung tissues was extracted and purified using Trizol reagent (Invitrogen, Carlsbad) according to the manufacturer’s protocol. RNA quantification and quality were measured by NanoDrop 1000 Spectrophotometer (Thermo, USA) and agarose gel electrophoresis as previously described [26, 27].

### Microarray analysis

The microarray hybridization was performed with service from KangChen Bio-tech (Shanghai, China), based on the manufacturer’s standard procedures. Briefly, mRNA was purified from 1 microgram of total RNA, and each sample was amplified and transcribed into fluorescent cRNA along the entire length of the transcripts without 3′ bias utilizing random primers. The labeled cRNAs were then hybridized onto the mouse lncRNA microarray V2.0 (8 × 60K, Arraystar). The arrays were then scanned by the Agilent Scanner G2565BA, and the analysis of array images was performed by Agilent Feature Extraction Software. Data normalization and subsequent processing were performed with the GeneSpring GX v12.1 software package (Agilent Technologies, Santa Clara, CA, USA).

Differentially expressed lncRNAs and mRNAs were identified by performing a volcano plot filtering, with the threshold defined as fold-change >2.0 (Student’s t-test *P* < 0.05) [28]. Hierarchical clustering was carried out to show the distinguishable lncRNA expression profile between CS-exposed mice and control samples.

### Independent validation of differentially expressed lncRNAs

Quantitative real-time PCR (qRT-PCR) was performed to validate the results of microarray analysis, and eight differentially expressed lncRNAs were randomly selected to be checked, primers for these lncRNAs were listed in Supplementary Table 1. Briefly, total RNA was extracted from the lung tissue as described above. cDNA was synthesized using the iScript cDNA Synthesis Kit (Bio-Rad, Hercules, CA, USA). qRT-PCR analysis was performed by the CFX96 real-time PCR detection system using SsoFast EvaGreen Supermix according to the manufacturer’s description (Bio-Rad, Hercules, CA, USA), and standard cure was used for the quantification of each lncRNA. Besides, all data were normalized to GAPDH gene expression. Differences in lncRNA expression between CS-exposed mice and controls were analyzed using Student’s t-test with SPSS 22.0 (SPSS Inc., Chicago, IL, USA), natural logarithm was calculated to analyze the relationship between fold changes of microarray analysis and qRT-PCR, two-side *P* < 0.05 was considered significant.

### GO and KEGG pathway analysis

We used Gene Ontology (GO) to predict the functions of differentially expressed genes identified in the present study, including molecular functions, biological processes, and cellular components. Pathway analysis was applied to map genes to Kyoto Encyclopedia of Genes and Genomes (KEGG) pathways. Fisher’s exact tests were also used for the statistical analyses.

### LncRNA-mRNA co-expression network

The coding-non-coding gene co-expression network (CNC network) was built according to the correlation between the differentially expressed lncRNAs and mRNAs. Pearson’s correlation coefficients (PCC) of no less than 0.9 were used to identify the lncRNA-mRNA pairs [29]. The lncRNA-mRNA co-expression network was drawn by Cytoscape software (The Cytoscape Consortium, San Diego, CA, USA).

## SUPPLEMENTARY MATERIALS TABLE


