# Supplementary material for: Microarray analysis of lung long non-coding RNAs in cigarette smoke-exposed mouse model
Source: Oncotarget. 2017 Dec 18;8(70):115647–56. doi: 10.18632/oncotarget.23362 (PMC5777800; doi:10.18632/oncotarget.23362)
Supplement: Supplementary file 1 [file oncotarget-08-115647-s001.pdf]

Microarray analysis of lung long non-coding RNAs in cigarette smoke-exposed mouse model

SUPPLEMENTARY MATERIALS

Supplementary Table 1: The primer sequences in this study

LncRNA Name	Forward primers (5'-3')	Reverse primers (5'-3')
uc011wph.1	CCGAGCCCAGGACTAAGAAA	ACTTCAGTTTCCTGTTCTTTCCA
AK076311	ACAGGACATCACTACACCCACG	GGAAGCCATACAAGCAAGAAGA
ENSMUST00000176356	TGGTAGTGCTGACAGCAACTGG	GCCAATGGTCACAGTCTGCTTA
uc008tgd.1	GAATGGTGGCAGTGGAATGT	TCATCCACATTTCGCACCC
uc007coi.2	CATCCGAACTACATCTGTTGAGC’	AGCCGTGAAGACCACAGAAAC
ENSMUST00000152414	GGCTGCCGTACAAAGTTCT	ACTGGGGATTCAACTCACG
ENSMUST00000155715	TGAGTGAGGTGGGAAATCG	CTAGACATGGGTGGGGAAA
ENSMUST00000181247	GTGGAGTGACTTGTCCTGAAATC	CAAATTCCTCCAGGACTTACGA
GAPDH(MOUSE)	CACTGAGCAAGAGAGGCCCTAT	GCAGCGAACTTTATTGATGGTATT
